# Breeding new seedless table grapevines for a more sustainable viticulture in Mediterranean climate

**DOI:** 10.3389/fpls.2024.1379642

**Published:** 2024-04-05

**Authors:** Luciana Piarulli, Costantino Pirolo, Vincenzo Roseti, Diana Bellin, Isabella Mascio, Pierfederico La Notte, Cinzia Montemurro, Monica Marilena Miazzi

**Affiliations:** ^1^ SINAGRI S.r.l. – Spin-Off of the University of Bari Aldo Moro, Bari, Italy; ^2^ Rete Italian Variety Club (IVC), Locorotondo, Italy; ^3^ Department of Biotechnology, University of Verona, Verona, Italy; ^4^ Department of Soil, Plant and Food Sciences, University of Bari Aldo Moro, Bari, Italy; ^5^ Istituto per la Protezione Sostenibile delle Piante (IPSP), CNR, Bari, Italy

**Keywords:** table grape, seedlessness, *Erysiphe necator*, *Plasmopara viticola*, resistance, marker-assisted selection

## Abstract

The growing demand for sustainable and environmentally friendly viticulture is leading to a multiplication of breeding programs aimed at obtaining vines that are resistant to powdery mildew (PM) and downy mildew (DM), the two most damaging vine diseases. In Puglia, the most important Italian region for the production of table grapes, an extensive crossing program was launched in 2015 with 113 crosses, including elite table varieties, seedless varieties, and resistant varieties. The main seedling production parameters were measured for each cross. In particular, berries harvested as well as the number of seeds and seedlings obtained were considered. Approximately 103,119 seedlings were obtained and subjected to marker-assisted selection for seedlessness using the marker VvAGL11 and for resistance to PM and DM with appropriate markers. Approximately one third (32,638) of the progenies were selected as putative seedless and seventeen thousand five hundred-nine (17,509) were transferred to the field for phenotypic evaluation, including 527 seedless individuals putatively resistant, of which 208 confirmed to be resistant to DM, 22 resistant to PM, and 20 individuals that combined resistance and seedlessness traits. The work discusses the effects of parental combinations and other variables in obtaining surviving progeny and pyramiding genes in table grapes and provides useful information for selecting genotypes and increasing the efficiency of breeding programs for seedless disease-resistant grapes.

## 1 Introduction

Italy is the fourth largest table grape producer in the world after China, Turkey, and Chile and ahead of the United States and South Africa, with a production of 1,005,552 million tons in 2021, and the main European reference in this sector (OIV, https://www.oiv.int/what-we-do/country-report?oiv, accessed June 19, 2023). In Italy, table grape production is localized in the Southern Italian regions, especially Apulia and Sicily, which are characterized by a hot and dry summer climate, mild winters with rainfall not exceeding 500–600 mm/year, and winter temperatures rarely below 0°C. Almost 60% of the Italian production is concentrated in Apulia, mainly with the varieties ITALIA, REGINA BIANCA, VITTORIA, MICHELE PALIERI, and RED GLOBE, which carry the protected geographical indication “Uva di Puglia I.G.P.” (UE Reg. n. 680/2012, 2012; 2020, ISTAT). Seedlessness and other traits, such as crispness, berry shape, color, and bunch compactness, have become highly valued by consumers (Somogyi et al., 2020). Since the commercialization of the variety Sultanina under the name Thompson Seedless in the 1970s (Anderson, 2013), many other varieties have been successfully developed and cultivated worldwide, such as SUGRAONE, CRIMSON SEEDLESS, and AUTUMN ROYAL. These varieties all carry steno-spermocarpic seedlessness, which is a highly heritable and dominant trait that can be selected very early using the intragenic microsatellite marker, *p3_VvAGL11*, mapped in the regulatory region of the VviAGL11 gene (Mejía et al., 2011; Bergamini et al., 2013; Royo et al., 2018; di Rienzo et al., 2020). However, these varieties are sold under Plant Breeders’ Rights protection, which limits their availability and increases costs. They also have adaptation problems in Apulia, which lead to browning of the skin and splitting of the berries and require special agronomic procedures (Otto et al., 2022). Another important quality parameter for table grapes is the color variation of the berry skin, which has become highly diversified through hybridization and human selection. Berry skin color is mainly related to the content and composition of anthocyanins, which is determined by the allelic status of an important locus spanning a 200-kb region on chromosome 2 (Azuma et al., 2008; Fournier-Level et al., 2009; Matus et al., 2009). In the last decade, several studies have shown that the R2R3-MYB gene is the major genetic determinant of anthocyanin content and composition in grape berry skin. However, further studies are needed to understand the complex network acting on this trait to fully understand and apply it in inbreeding programs.

The control of diseases by genetic resistance sources, in particular powdery mildew (PM) and downy mildew (DM), caused by the biotrophic fungi *Erysiphe necator* (Schwein.) Burrill and *Plasmopara viticola* (Berk. & M.A. Curtis) Berl. and De Toni, respectively, has become necessary to obtain high-quality grape productions. The former usually attacks in warm and dry seasons, while the latter disease occurs in warmer and more humid climates (Miazzi et al., 1997; Hajjeh et al., 2008; Fröbel and Zyprian, 2019). Both diseases require a massive usage of pesticides, which is associated with high economic and environmental costs, including the emergence of resistance in pathogens (Hajjeh et al., 2005; Miazzi et al., 2008; Pedneault and Provost, 2016). The strict regulation of pesticides (Directive 2009/128/EC; Regulation 2002/473/EC) and the increasing demand for organic products have made sustainability an important issue in grape cultivation. Efforts to develop more sustainable viticulture have multiplied prompting private and public organizations to expand the use of resistant varieties introgressing genes from American non-*vinifera* species (such as *Vitis riparia*, *Vitis rupestris*, *Muscadinia rotundifolia*, *Vitis cinerea*, *Vitis berlandieri*, *Vitis lincecumii*, *Vitis labrusca*), Asian species (*Vitis piasezkii*, *Vitis amurensis*, *Vitis romanetii*), and *Vitis vinifera Kishmish vatkana*. These species have been used in breeding programs for decades (Bellin et al., 2009; Venuti et al., 2013; Feechan et al., 2015; Ibáñez et al., 2015; Ruehl et al., 2015; Merdinoglu et al., 2018; Vezzulli et al., 2018; Bove et al., 2019; Pomarici and Vecchio, 2019; Weinmann et al., 2019; Possamai et al., 2022; Schneider et al., 2019; Karn et al., 2021). After the first poor-quality hybrids, new resistant varieties, such as Regent or Solaris, have met the quality standards of the European market [30–32] (Töpfer et al., 2011, Buonassisi et al., 2017) paving the way for a wider use of genotypes coming from interspecific crosses also in the competitive table grape breeding programs, together with additional traits such as seedlessness. According to the VIVC database (https://www.vivc.de/index.php), 35 loci for downy mildew resistance (*Rpv* loci) and 13 loci for powdery mildew resistance (*Run* and *Ren* loci) have been mapped so far and are available for breeding even if only a few of them have been used so far. Associated markers are available for these loci, enabling marker-assisted selection (MAS) of resistant individuals at early stages of plant development (Eibach et al., 2007; Pathania et al., 2017; Cobb et al., 2019; Vezzulli et al., 2019; Zini et al., 2019), thus, greatly facilitating breeding programs.

This is particularly true for table grapes, where fungicides have to be applied repeatedly since early spring and throughout the growing season (Miazzi and Hajjeh, 2011; Chen et al., 2020). Over the last 20 years, developments in biotechnology and molecular biology have provided breeders with useful tools to speed up programs and make them more efficient. Genotyping and traceability methods based on molecular markers (Di Rienzo et al., 2017; Miazzi et al., 2020; Sanzani et al., 2016), and next-generation sequencing (NGS) technologies have led to more innovative and targeted studies in grape breeding (Butiuc and Coste, 2023; Vervalle et al., 2022). The intensive chemical treatments required to control them imposes expensive costs on growers and the environment, contribute significantly to pollution, and ultimately lead to a decline in the effectiveness of pesticides (Reynolds, 2015; Lykogianni et al., 2021).

This paper presents the results of a five-year breeding program carried out since 2015 by the Apulian winegrowers’ association “Italian Variety Club” (IVC, Bari, Italy) to obtain new grape varieties that combine seedlessness with resistance to PM and DM. The analysis of parental combinations/cultivation conditions for the development of new hybrids and an evaluation of the success of pyramidization will provide useful information to improve the efficiency of breeding seedless resistant grape varieties.

## 2 Material and methods

### 2.1 Plant material

For the crossing program, 38 stenospermocarpic seedless elite table grape varieties and pre-breeding material, and 42 elite seeded table varieties were selected (**Table 1**). Among them, seven were resistant to DM and PM, deriving from the species *V. riparia*, *V. rupestris* (Vitis International Variety Catalogue, VIVC, https://www.vivc.de/), and possibly *M. rotundifolia.* The identity of the parental varieties was preliminarily confirmed by PCR amplification with nine SSR markers established by the OIV for genotyping grapevines (OIV, accessed 10/5/2023) [36], according to the VIVC and DISSPA databases (**Supplementary Table 1**). All vines were 10 years old and were grown either at the Experimental Station “Centro di Ricerca e Formazione Basile Caramia” in Locorotondo (Bari, Italy) or at the IVC producer’s consortium (Bari, Italy) at a planting density of 1.0 × 2.5 m trained to a T-trellis. The vines were covered with a thin white net, and 0.16-mm-thick white polyethylene was applied from stage BBCH-11 (Lorenz et al., 1995). Irrigation was provided every 3 to 4 days by a drip irrigation system to keep the soil above 75% of field capacity. All agronomic practices were applied uniformly in all treatments and were consistent with standard commercial practice in the area.

**Table 1:** List of *V. vinifera* cultivars used for the cross combinations between seeded (SD, female parent) and seedless (SL, male parent) cultivars carried out from 2015 to 2019.

Cross code	Year	Code	Seeded variety			Seedless variety		
			Name	Color	Flavor	Name	Color	Flavor
1	2019	DISSPA-UNIBA	Aabaidi	White	Neutral	AP29	White	Muscat
2	2016	VIVC 42205	Alicante	Black	Neutral	AP29	White	Muscat
3	2016					AP21	White	Muscat
4	2016					AP34	Red	Neutral
5	2016	VIVC 20930	Barbarossa	Red	Neutral	AP4	White	Neutral
6	2018	VIVC 10171	Baresana	White	Neutral	AP30	White	Neutral
7	2018					AP29	White	Neutral
8	2019					AP7	White	Muscat
9	2018	VIVC 987	Baresana rosa	Red	Neutral	AP28	Red	Muscat
10	2016	DISSPA-UNIBA	Beccarosa	Red	Neutral	AP33	Red	Neutral
11	2017					AP6	Red	Neutral
12	2015	VIVC 7569	Black Magic	Black	Neutral	AP33	Red	Neutral
13	2016					AP36	Black	Neutral
14	2017	VIVC 1404	Black Pearl	Black	Neutral	AP18	Black	Neutral
15	2017					AP28	Red	Muscat
16	2017	DISSPA-UNIBA	Bolgar Rezy*	White	Neutral	AP23	White	Neutral
17	2017					AP4	White	Neutral
18	2018					AP19	White	Muscat
19	2018					AP29	White	Muscat
20	2018					AP8	White	Muscat
21	2017	VIVC 2091	Cardinal	Red	Neutral	AP29	White	Muscat
22	2017					AP36	Black	Neutral
23	2017					AP1	Red	Neutral
24	2017					AP11	White	Muscat
25	2017					AP28	Red	Muscat
26	2018					AP28	Red	Muscat
27	2015	VIVC 2724	Corniola	White	Neutral	AP17	White	Neutral
28	2016					AP22	Black	Muscat
29	2016					AP25	White	Neutral
30	2017					AP33	Red	Neutral
31	2017					AP32	White	Neutral
32	2019	DISSPA-UNIBA	Corniola rosa	Red	Neutral	AP7	White	Aromatic
33	2015	VIVC 122	Dattero	White	Neutral	AP13	White	Neutral
34	2015					AP29	White	Muscat
35	2018	VIVC 3904	Emperor	Red	Neutral	AP28	Red	Muscat
36	2019	DISSPA-UNIBA	Ignota ibrido f23pb	White	Neutral	AP18	Black	Neutral
37	2017	VIVC 23008	Guzun*	White	Neutral	AP29	White	Muscat
38	2017					AP9	Red	Neutral
39	2017					AP4	White	Neutral
40	2017					Italia	White	Muscat
41	2018					AP29	White	Muscat
42	2019					AP9	Red	Neutral
43	2019					AP19	White	Muscat
44	2019					AP29	White	Muscat
45	2018	DISSPA-UNIBA	Hifavi	White	Neutral	AP30	White	Neutral
46	2015	VIVC 5582	Italia	White	Neutral	AP24	Black	Neutral
47	2015					AP29	White	Muscat
48	2016					AP25	White	Neutral
49	2016					AP4	White	Neutral
50	2017					AP23	White	Neutral
51	2017					AP1	Red	Neutral
52	2017					AP9	Red	Neutral
53	2018					AP28	Red	Muscat
54	2018					AP30	White	Neutral
55	2018					AP3	White	Neutral
56	2018					AP1	Red	Neutral
57	2018					AP7	White	Aromatic
58	2017	DISSPA-UNIBA	Italia-2	White	Neutral	AP15	White	Neutral
59	2017					AP29	White	Muscat
60	2017					AP31	Red	Neutral
61	2018	DISSPA-UNIBA	Italia CRSFA 121	White	Neutral	AP30	White	Neutral
62	2019	DISSPA-UNIBA	Lattuario francese	White	Neutral	AP29	White	Neutral
63	2018	VIVC 6771	Lattuario nero	Black	Neutral	AP28	Red	Muscat
64	2016	VIVC 24820	Mennavacca	White	Neutral	AP25	White	Neutral
65	2019					AP23	White	Neutral
66	2017	VIVC 7896	Moldova*	Black	Neutral	AP5	Black	Neutral
67	2017					AP1	Red	Neutral
68	2016	VIVC 8226	Moscato d’Amburgo	Black	Neutral	AP33	Red	Neutral
69	2016					AP32	White	Neutral
70	2017					AP2	Black	Neutral
71	2018					AP19	White	Muscat
72	2016	VIVC 8050	Moscato d’Adda	Black	Neutral	AP25	White	Neutral
73	2016					AP25	White	Neutral
74	2016	VIVC 8056	Moscato giallo	White	Neutral	AP13	White	Neutral
75	2019	VIVC 8210	Muscat Saint Vallier*	White	Neutral	AP19	White	Muscat
76	2019					AP29	White	Muscat
77	2015	VIVC 8716	Ohanez	White	Neutral	AP7	Red	Neutral
78	2018					AP3	White	Neutral
79	2019					AP23	White	Neutral
80	2018	VIVC 14012	Palatina*	White	Neutral	AP29	White	Muscat
81	2019					AP29	White	Muscat
82	2017	VIVC 7704	Michele Palieri	Black	Neutral	AP18	Black	Neutral
83	2017					AP36	Black	Neutral
84	2017	VIVC 16448	Pizzutella	White	Neutral	AP23	White	Neutral
85	2017					AP35	Black	Neutral
86	2017					AP10	White	Aromatic
87	2017					AP14	White	Neutral
88	2019	VIVC 8207	Poloskei muskotaly*	White	Neutral	AP9	Red	Neutral
89	2017	VIVC 9707	Primus	White	Neutral	AP8	White	Muscat
90	2016	VIVC 9972	Red Globe	Red	Neutral	AP16	White	Muscat
91	2016					AP22	Black	Muscat
92	2016					AP7	Red	Neutral
93	2017					AP20	Red	Muscat
94	2018					AP3	White	Neutral
95	2018	DISSPA-UNIBA	Red Italy	Red	Neutral	AP28	Red	Muscat
96	2019					AP33	Red	Neutral
97	2019					AP9	Red	Neutral
98	2019	DISSPA-UNIBA	S. Anna nera	Black	Neutral	AP33	Red	Neutral
99	2019	DISSPA-UNIBA	Sacra rossa	Red	Neutral	AP23	White	Neutral
100	2019	DISSPA-UNIBA	Sacra rossa 2	Red	Neutral	AP12	Black	Aromatic
101	2018	VIVC 11932	Souri	White	Neutral	AP30	White	Neutral
102	2019					AP29	White	Muscat
103	2017	VIVC 14323	Terez*	White	Neutral	AP33	Red	Neutral
104	2018					AP29	White	Muscat
105	2019					AP9	Red	Neutral
106	2016	13031	Vittoria	White	Neutral	AP29	White	Muscat
107	2017					AP29	White	Muscat
108	2017					AP36	Black	Neutral
109	2018					AP29	White	Muscat
110	2016	VIVC 23008	Guzun	White	Neutral	AP28	Red	Muscat
111	2016	VIVC 14012	Palatina	White	Neutral	AP33	Red	Neutral
112	2016	VIVC 8207	Poloskey	White	Neutral	AP32	White	Neutral
113	2016	VIVC 14323	Terez	White	Neutral	AP4	White	Neutral

Varieties resistant to DM and PM according to their pedigree (VIVC) or previous literature reports are marked with an asterisk (*).

### 2.2 Crosses and seeds recovery

From 2015 to 2019, a total of 113 crosses was made. A single variety was used in combination with several different varieties to compare the crossing performances of the different combinations (**Table 1**). Most of the cultivated grapes are hermaphroditic, and fertilization occurs mainly by self-pollination (Harst et al., 2009). In programmed crosses, therefore, the emasculation of plants to be used as female parents is necessary. Late-seeded varieties were chosen as female. Pollen was collected from fully flowering vines (at least 30%) at stages BBCH-65 and BBCH-68, while the female parent was used at stage BBCH-60 (first flower hoods detached from the *receptaculum*) (Lorenz et al., 1995). Pollen was collected avoiding humid, windy, and rainy conditions and stored at 18°C. Before use, pollen germinability was tested on paper in Petri dishes as percentage of granules producing germination tubes; samples with a germination of <50% were discarded.

**Figure 1:**
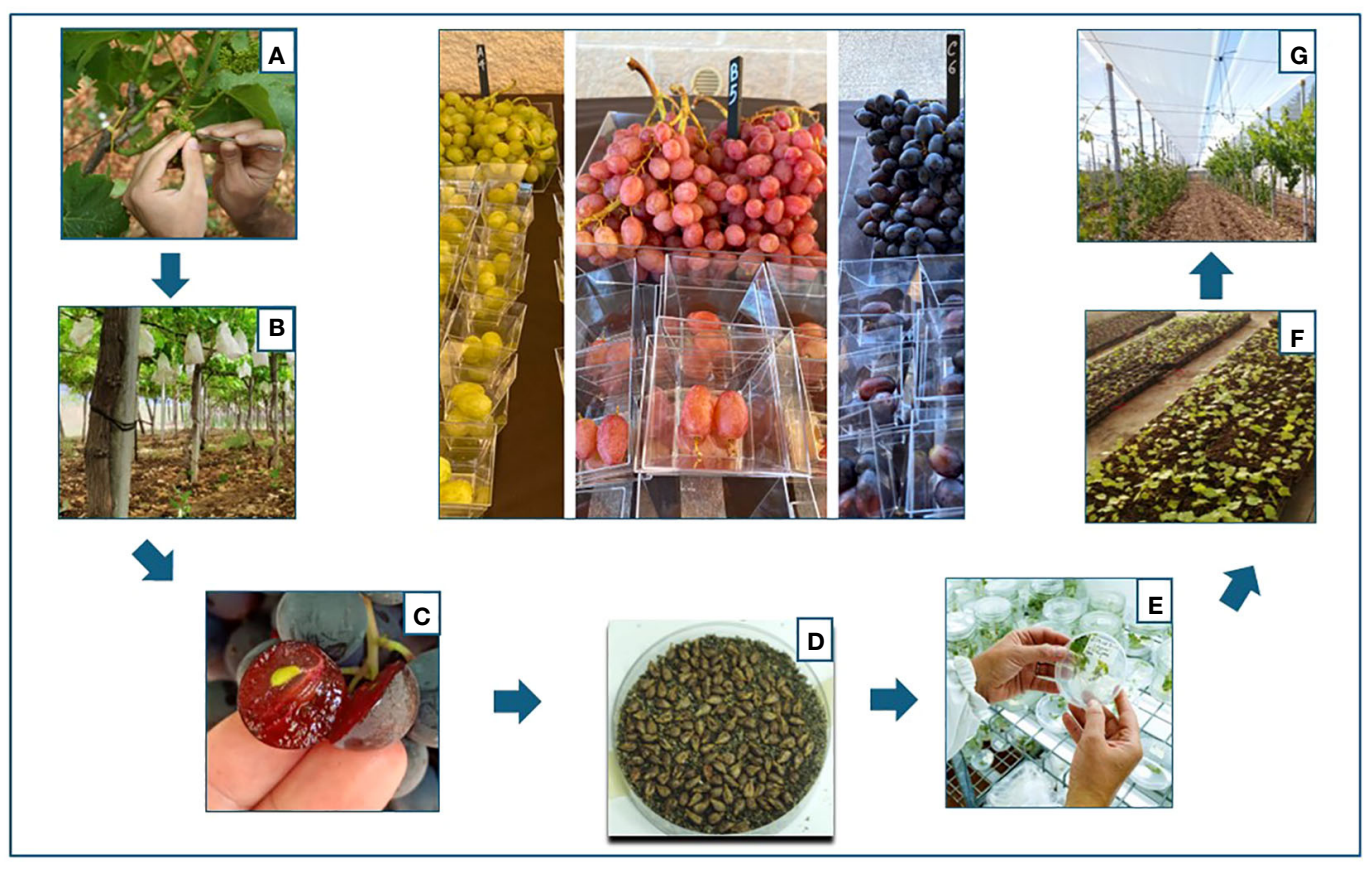
Flowchart of the breeding process followed to obtain the new varieties: **(A)** emasculation of the flowers of the female parents; **(B)** pollination and casing of the bunches with paper bags; **(C)** collection of the seeds; **(D)** stratification of the seeds in sand for vernalization; **(E)** germination of the seeds; **(F)** transfer and acclimatization of the vines in the greenhouse; **(G)** selection of the vines in the field.

A total of 1,612 bunches were pollinated. The inflorescences were emasculated with sterile tweezers and pollinated twice with a sterile brush within 48 h. The pollinated inflorescences were immediately packed until complete berry development. After first fruit development (10 days), bags were removed to allow regular growth of the grapes. Depending on the combination, fully ripe grapes with completely woody seeds were harvested from the end of August through September. The seeds were extracted from the ripe berries, washed in 1% hypochlorite solution, rinsed three times in sterile distilled water, and dried on paper at 25°C and low humidity for approximately 4–5 days until they were completely dry. Then they were stored in Petri dishes in the dark, under cool and dry conditions (4°C) for vernalization (Wang et al., 2022) (**Figure 1**).

### 2.3 Seed vernalization, germination, and plant development

Seeds were vernalized by stratification in thin, moistened river sand autoclaved at 120°C for 40 min. Seeds were stored in the dark at 4°C for approximately 1.5 months taking care not to let it mold or rot. For some varieties, this phase was extended to at least 3 months. After vernalization, seeds were transferred to paper discs in Petri dishes for germination, then transplanted into paper pots (250 ml) containing a soil mixture of vermiculite/peat soil/coconut shells (1:4:1) and placed in a greenhouse at 24°C and natural daylight for 1 month for acclimatization, after which they were transplanted into the open field (**Figure 1**).

### 2.4 DNA extraction and marker-assisted selection

Two young leaves were taken from each plantlet and used for DNA extraction following the protocol of Spadoni et al. (2019). The quantity and quality of extracted DNA was measured using the Nano-Drop™ 12000C spectrophotometer (Thermo Scientific, Waltham, MA) and 0.8% agarose gel electrophoresis. Seedlessness was selected using the SSR marker VvAGL11 with forward primer labeled with a 6-FAM, HEX, NED fluorescent dye (Bergamini et al., 2013). A final PCR volume of 12.5-μl mixture was used, including 2 ng/µl of DNA, dNTP 0.4 mM, 1.25 µl of PCR buffer 2×, 1.25 µl of primer mix forward and reverse (2.5 ng/µl), 0.1 U of DreamTaq polymerase. Reactions were performed under the following conditions: 95°C, 5 min; 10 cycles: 95°C, 30 s; 55°C, 45 s; 72°C, 45 s with a touch-down of 0.5°C per cycle; 25 cycles: 95°C, 30 s; 50°C, 45 s; 72°C, 45 s; final extension to 72°C, 15 min.

For resistance selection, a preliminary screening of the resistance genes present in the parents used for the crosses was carried out, as they were provided by Novisad University (Serbia), and the pedigrees of many of them were unknown. Therefore, four SSR markers associated with the *Rpv1* and *Rpv3* genes for DM and five SSR markers associated with the *Run1*, *Run2*, *Ren1*, and *Ren4* genes for PM were selected based on a bibliographic review (VIVC, Di Gaspero et al., 2012; Li et al., 2012; Prazzoli et al., 2019; Zini et al., 2019) (**Table 2**). A preliminarily validation of markers on the resistant parents was carried out and for four among the resistant genotypes we got so far, robust and effective amplifications with markers UDV305 and UDV737 for DM, and VMC4f3.1 for PM giving the expected resistant allele profiles. Thus, these crosses were retained for subsequent MAS analysis in progenies (**Table 3**). PCRs were carried out in a final volume of 20 μl using the following concentrations: 2 ng/µl of DNA, dNTP 0.4 mM, PCR buffer 2×, 1 µl of primer forward (1 ng/µl), 3.2 µl of primer reverse (1 ng/µl), 1.6 µl of universal primer M13(-20) (1 ng/µl) labeled with a 6-FAM, HEX, NED fluorescent dye, and 0.25 U of DreamTaq polymerase. The following conditions were used: 95°C, 2 min; 10 cycles: 94°C, 20 s; 55°C, 20 s; 65°C, 40 s with a touch-down of 0.5°C per cycle; 25 cycles: 94°C, 20 s; 50°C, 20 s; 65°C, 40 s; final extension to 65°C, 30 min. Capillary electrophoresis was performed using the ABI PRISM 3100 Genetic Analyzer (Life Technologies) mixing 2 µl of the amplification products with 14.6 µl of formamide and 0.5 µl of the GeneScan 500 ROX size standards (Life Technologies, Carlsbad, CA, USA). Allele sizes were assigned using GeneMapper^®^ software version 3.7 (Life Technologies).

**Table 2:** List of markers selected for the analysis of loci associated with resistance (R) to DM and PM and used for a pre-screening on resistant parentals.

Resistance	Locus	Chr	Associated marker	Resistance allele (bp)	Genotype of origin	Reference
***Plasmopara viticola***	*Rpv1*	12	VMC1g3.2	122	VRH30-82-1-42 (*V. vinifera* × *M. rotundifolia*)	Zini et al., 2019
				118		Prazzoli et al., 2019
	*Rpv3*	18	VMC7f2	210	*(V. rupestris)*	Prazzoli et al., 2019
	*Rpv3 ^(Rpv3 321-312)^*	18	UDV305	321	Chancellor	Zini et al., 2019
			UDV737	312	Noah (*V. labrusca, V. riparia*)	Di Gaspero et al., 2012
	*Rpv3 ^(Rpv3 361-299)^*		UDV305	361	Villard blanc (*V. rupestris*)	Zini et al., 2019
			UDV737	299	Ganzin (*V. rupestris*)	Di Gaspero et al., 2012
	*Rpv3.1 ^(Rpv3 299-279)^*	18	UDV305	299	Villard blanc (Seibel) (*V. rupestris*)	Zini et al., 2019
			UDV737	279	Seibel 4614 (*V. rupestris*)	Di Gaspero et al., 2012
	*Rpv3.2 ^(Rpv3 null-297)^*	18	UDV305	Nulli	Seibel/Seyval (*V. rupestris*)	Zini et al., 2019
			UDV737	297	Munson (*V. rupestris*)	Di Gaspero et al., 2012
	*Rpv3.3 ^(Rpv3 null-271)^*	18	UDV305	Nulli	Seyval (*V. rupestris*)	Zini et al., 2019; Foria et al., 2015
			UDV737	271	Noah (*V. labrusca o V. riparia*)	Di Gaspero et al., 2012; Foria et al., 2018
***Erysiphe necator***	*Run1*	12	VMC8g9	160	VRH3082-1-42 (*V. vinifera x M. rotundifolia*)	Zini et al., 2019
				159	NC6-15 (*V. rotundifolia*)	Riaz et al., 2011
				156		Prazzoli et al., 2019
			VMC4f3.1	186	VRH3082-1-42 (*M. rotundifolia*)	Yıldırım et al., 2019
				182	Trayshed2 (*M. rotundifolia)*	Riaz et al., 2011
				188	*V. rotundifolia*	Prazzoli et al., 2019
				192	NC6-15 (*V. rotundifolia*)	Riaz et al., 2011
	*Run2.1*, *Run2.2*	18	VMC7f2	195	Trayshed *(M. rotundifolia)*	Riaz et al., 2011; Zini et al., 2019
				193	Magnolia (*M. rotundifolia*)	
	*Ren1*	13	UDV124	214	Kishmish vatkana (*V. vinifera*)	Prazzoli et al., 2019; Hoffmann et al., 2008
	*Ren4*	18	UDV108	220	Trayshed *(M. rotundifolia)*	Ramming et al., 2011; Riaz et al., 2011
				202	Magnolia (*M. rotundifolia*)	

Locus, chromosome, resistance allele/haplotype, genotype of origin, and bibliographic reference are indicated.

**Table 3:** Profiles of resistance obtained on parental varieties to confirm the presence of loci of resistance to DM and PM.

Variety	DM resistance-associated gene				PM resistance- associated gene					
	*Rpv3.1*				*Run 1*		*Run2.2*		*Ren4*	
	UDV305		UDV737		VMC4f3.1		VMC7f2		UDV108	
Palatina	**299**	343	**279**	285	166	174	124	134	–	242
Poloskey	**299**	299	**279**	285	164	174	124	124	242	242
Terez	**299**	343	**279**	285	166	**182**	124	134	**220**	242
Guzun	**299**	327	**279**	295	174	**182**	124	134	234	242

Bold indicates expected alleles; hyphens indicate the null alleles.

### 2.5 Evaluation of selected vines in the field

The selected progenies were propagated in 2016 and evaluated in a comparison field made in 2017 using 24 plants for each genotype according to the comparison criteria required for registration (Ministerial Decree n. 489243, Ministro delle Politiche Alimentari e Forestali, 30/09/2021). Vines were grown in the field for 3 years and then phenotyped in the field for seedlessness and for the important commercial traits of grape color and aroma. Putative seedless vines were transplanted into the field for phenotypic trait evaluation on each vine. After 3 years, seedlessness was assessed on 100 berries per vine according to Bergamini et al. (2013) and a three-class seed scale: aborted or vestigial (C1), herbaceous (C2), and woody (C3). Resistance to DM and PM was assessed during the highest disease pressure using the susceptible variety Italia as a control. Each vine was examined for symptoms classified according to the OIV455-1 (OIV, 1984) on a scale from 0 to 9 as follows: 1. Very low resistance: leaf with dense sporulation over the entire leaf surface and on more than 75% of the plants. 3. Low resistance: dense sporulation over the 65%–100% of the leaf surface, 50%–75% of the plants affected. 5. Medium resistance: sporulation over the 25%–65% of the leaf surface, 36%–50% of the plants affected; 7. Strong resistance: scant sporulation over the 5%–25% of the leaf surface, 25%–35% of the plants affected; 9. Very strong resistance: sporulation on 0%–5% of the leaf surface, <25% of the plants affected. On all the progenies, “berry color” (white, pink, red, black, neutral) and “berry flavor” (aromatic, neutral foxy) were also noted.

## 3 Results

### 3.1 Crosses

During the 5-year program, 109 crosses were successfully carried out by pollinating seeded varieties with seedless varieties yielding a total of 121,723 seeds. An average of 1,077 seeds per cross were obtained, ranging from 90 (Italia × AP28) to 4,600 (Red Globe × AP20). This combination also had the highest number of seeds per berry (2.6; average 1.5) (**Supplementary Table 2**).

### 3.2 Seed vernalization, germination, and plant development

**Figure 2:**
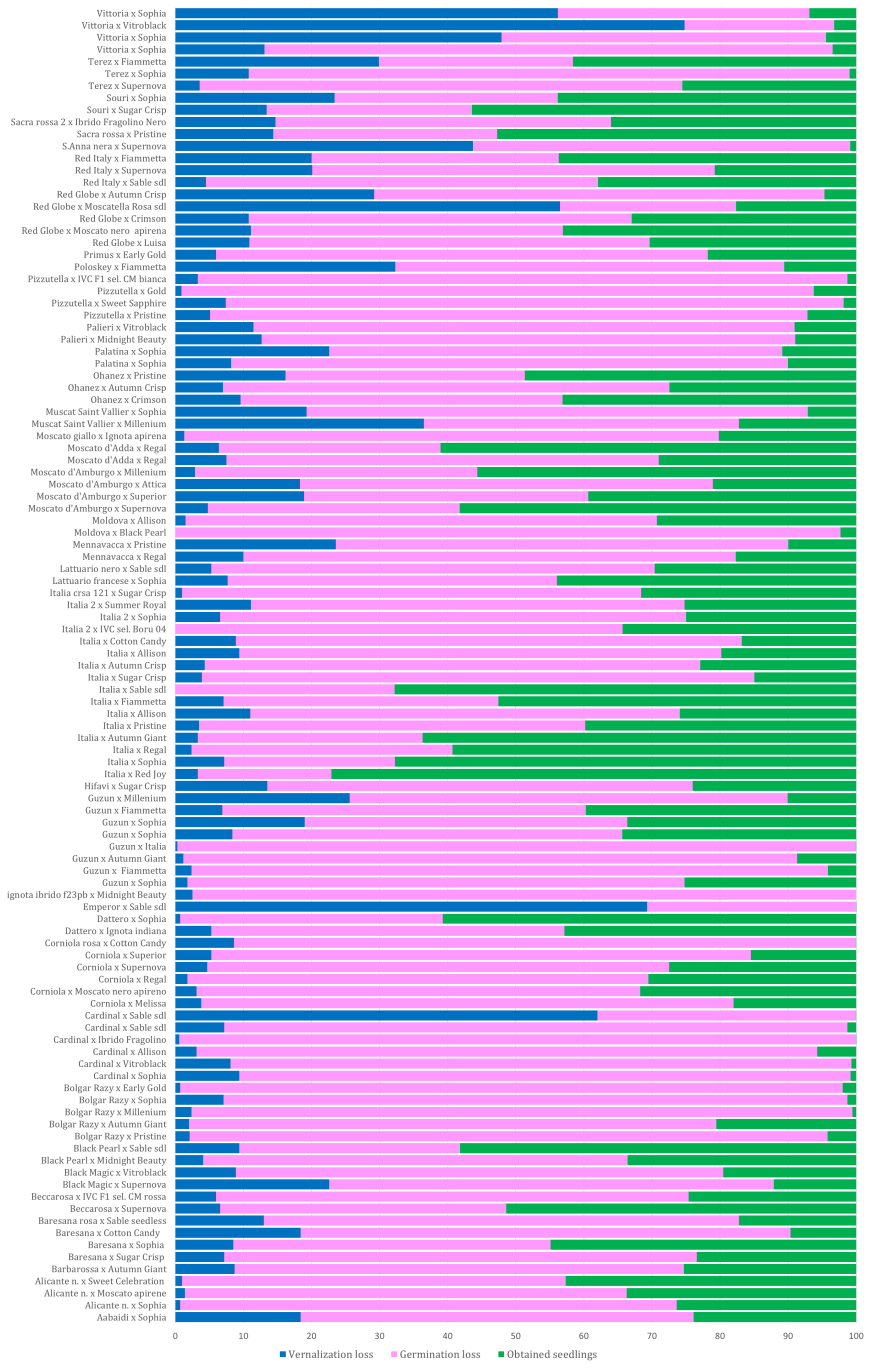
Percentage of seed loss observed during vernalization and germination for each cross; the percentage of viable seedlings transferred to the greenhouse is shown in green. The squares with a solid line indicate some of the best crosses; the dotted squares indicate some of the worst crosses. Overall, the crosses with cultivars Victoria and Red Globe showed losses >50%, while very low losses were observed in the progenies of cultivars Italia (× AP24, 3.3%; × AP23, 3.5%; × AP30, 3.9%; × AP29, 7.2%; × AP25, 2.4%; × AP4, 3.3%) as well as cultivars ALICANTE, BOLGAR RAZY, CORNIOLA, and GUZUN (**Supplementary Table 2**; **Figure 2**).

All collected seeds were sent for vernalization during which some die-offs occurred. During vernalization, the average loss was 12.3%, but very high losses were observed in cross Victoria × AP36 (74.8), Cardinal × AP28 (62%), and Emperor × AP28 (69.3%) (**Supplementary Table 2**; **Figure 2**).

A total of 103,119 seeds were brought into the greenhouse for germination and acclimatization. During these stages, a further average loss of 62% was observed reducing the number of seedlings to 32,638 (**Supplementary Table 2**). The highest losses were observed in the progeny of varieties, such as CARDINAL, BOLGAR RAZY, MOLDOVA, PIZZUTELLA, and Victoria, as female plants. In contrast, the lowest losses were observed in progenies of ITALIA, SOURI, SACRA ROSSA, RED ITALY, RED GLOBE, DATTERO, MOSCATO D’AMBURGO, among others (see **Figure 2**). Progenies from the crosses BOLGAR RAZY × AP19, GUZUN × ITALIA, IGNOTA IBRIDO F23PB × AP18, CORNIOLA ROSA × AP7, and EMPEROR × AP28 were cleared (**Supplementary Table 2**; **Figure 2**). An effect of the combination “female × pollen donor” was observed in the percentage of progeny surviving in the different developmental stages. For example, a few progenies from crosses of Italia with AP1, AP7, and AP30 survived, while approximately 70% of the progeny from crosses with AP4, AP24, and AP28 survived with a small loss during the germination stage. Losses in the greenhouse and in the field were negligible, as good crop protection and cultivation practices were applied.

### 3.3 Molecular analysis of seedlessness

Molecular analysis of seedlessness was carried out on 60 selected crosses of the program, including four crosses for resistance to DM and PM. A total of 17,509 individuals were tested with the marker p3_VvAGL11 associated with the major seedlessness gene identifying 8,223 putative seedless individuals corresponding to 47% of the total progeny (**Supplementary Table 3**). This, matched with the expected segregation rate of 1:1 for crosses between seeded parents (woody seeds, homozygous at the *p3_VvAGL11* locus, allele profile *184/184* bp) × seedless parents (herbaceous or aborted seeds, heterozygous at the *p3_VvAGL11* locus, allele profile *184/196* bp) resulted in genotypes *184/184* (seeded) and 184/196 (seedless or herbaceous seeds) (Bennici et al., 2019). However, large deviations from the expected values were also observed, such as the 3% in the cross BECCAROSA × AP33 and the 87% in the cross PIZZUTELLA × AP23, as well as in crosses with the varieties CORNIOLA and VITTORIA (**Supplementary Table 3**). The seedlessness obtained also varied greatly within the crosses of the female variety in relation to the pollinator variety, as, for example, in the crosses of cv Italia where seedless progeny ranged from 31% in the cross with AP7 to 80% in the cross with AP30.

### 3.4 Molecular analysis of the resistance

The 527 seedless individuals obtained from the four crosses, including a resistant variety as parent, were subjected to MAS for resistance too. The haplotype *299-279*, associated with the DM resistance gene *Rpv3.1*, was found in a total of 206 individuals, while the allele *182*, associated with the PM resistance gene *Run1*, was observed in 22 individuals (**Table 4**; **Supplementary Table 4**). None of the resistant progeny to PM was obtained in crosses PALATINA × AP33 and POLOSKEY × AP32, while pyramidization of the two resistances was achieved in 20 individuals obtained from the crosses GUZUN × AP28 and TEREZ × AP4 (**Table 4**; **Supplementary Table 4**).

**Table 4:** Summary of the results obtained in the grapevine cross-program for pyramidation of seedlessness and resistance to DM and PM.

Cross code	Seeded resistant variety	Seedless susceptible variety	Number of obtained progeny	Number of seedless progeny	Number of DM resistant progeny (*Rpv3 ^299-279^*)	Number of PM resistant progeny (*Run1^182^*)	Number of DM and PM resistant progeny
110	Guzun	AP28	270	135	59	7	5
111	Palatina	AP33	193	98	41	0	0
112	Poloskey	AP32	218	94	34	0	0
113	Terez	AP4	428	200	72	15	15
Total			1,109	527	206	22	20

### 3.5 In-field phenotypic evaluations

**Figure 3:**
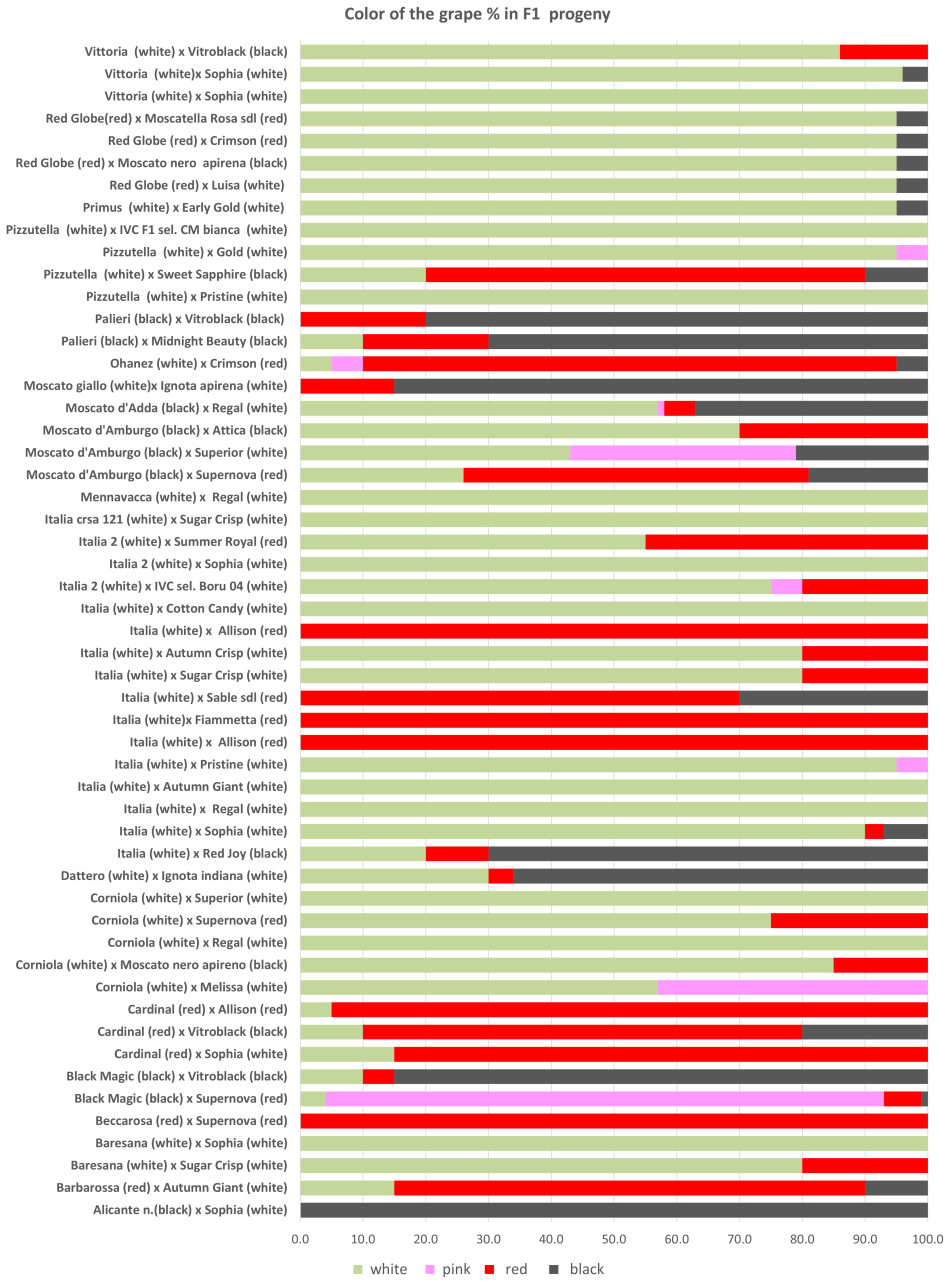
The percentages of genotypes with different berry color observed in F1 progenies obtained in the crosses.

The progenies selected just for seedlessness were propagated in 2016 and evaluated in a comparison field made in 2017 using 24 plants for each genotype according to the criteria required for registration (see Material and Methods). Overall, seedlessness was confirmed in 79% of the vines, while 21% of the vines carried herbaceous (C1) or woody seeds (C3). In particular, herbaceous seeds (C1) were observed in the progeny of ITALIA × AP29 (14%), MENNAVACCA × AP25 (9%), PIZZUTELLA × AP23 (10%), and PIZZUTELLA × AP35 (15%), and woody seeds (C3) were found in the progeny of BLACK MAGIC × AP33 (31%), BLACK MAGIC × AP36 (6%), CORNIOLA × AP17 (26%), CORNIOLA × AP25 (52%), ITALIA × AP24 (13%), MENNAVACCA × AP25 (26%), MOSCATO D’ADDA × AP25 (37%), and MOSCATO GIALLO × AP13 (36%) (**Supplementary Table 3**). As for color, the breeding program produced 55.1% white, 3.6% pink, 24.6% red, and 4.1% black vines (**Supplementary Table 5**), with recombination occurring in 34 out of 56 crosses (**Figure 3**), particularly in crosses with BLACK MAGIC, MOSCATO, and RED GLOBE.

**Figure 4:**
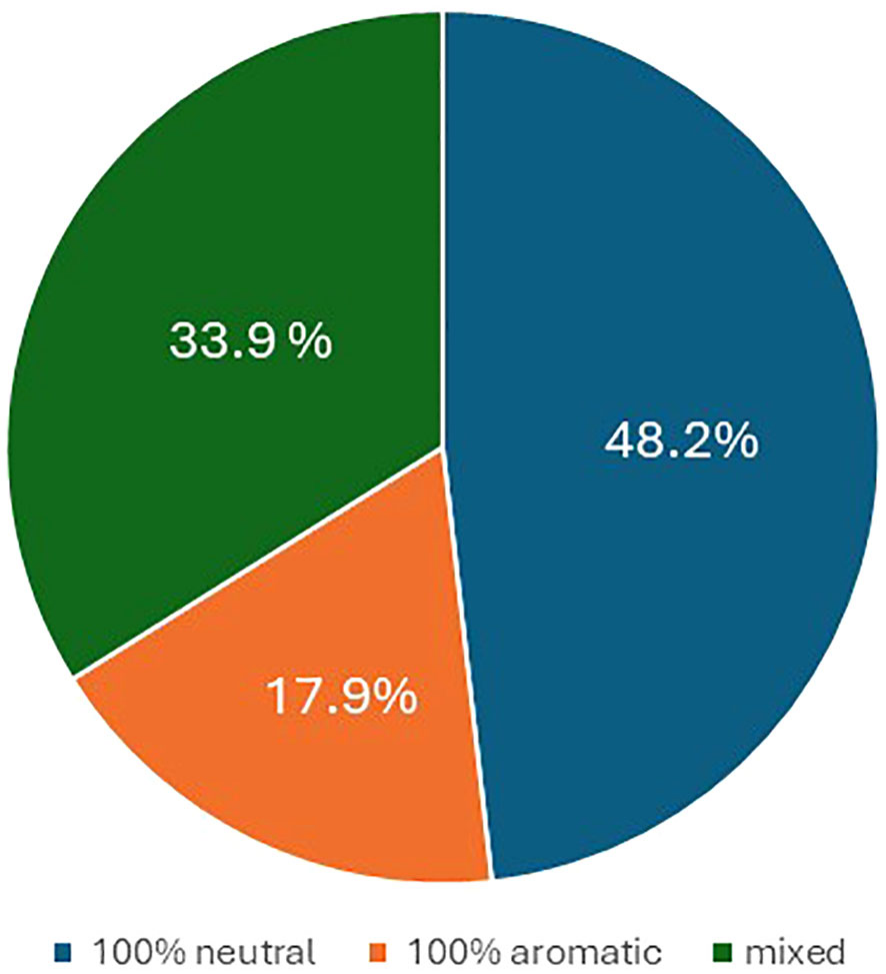
Pie chart illustrating the percentage of progenies with berry flavor neutral (blue), aromatic (orange), and mixed (gray).

In terms of flavor, 48.2% of the offspring were neutral, 17.9% were aromatic, while the remaining 33.9% were partly aromatic and partly neutral (**Figure 4**).

The seedless progenies of the crosses also selected for DM and PM resistance were scored in the field during the peak disease pressure using a scale from 0 to 9. The results generally confirmed the molecular data for both DM and PM and showed medium-high score on the resistance scale, although some contradictory data were also found (**Supplementary Table 4**). Further investigation is needed both in molecular screening with more markers and markers for additional loci and in the field for symptoms. Indeed, the absence of R loci stands solely for the loci analyzed and leaves open the possibility that other markers may be useful for detecting additional sources of resistance.

## 4 Discussion

In Apulia, table grapes are of crucial economic importance, and growers demand good table grape varieties that are adapted to the local climate and combine seedlessness with resistance to destructive diseases such as powdery mildew and downy mildew. To meet these expectations, the Apulian winegrower’s association “Italian Variety Club” launched an extensive crossing program in 2015 to combine seedlessness with durable resistance in new grape varieties. The plan comprised 113 crosses, which included seedless and elite varieties as parents, as well as four varieties with resistance to PM and DM diseases. A total of 121,723 seeds were collected with the crossing program, but vernalization resulted in an average loss of 12.1% of the seeds, which proved to be a very critical phase. Nevertheless, the results were encouraging considering that the germination capacity of grapevine seeds is generally approximately 30%–50% (Gao et al., 2014; Wang et al., 2022). Seed germination is one of the most important determinants of reproductive performances and depends on various factors [8], such as stratification time, amount of moisture, mold development, amount of inhibitory phenolic compounds, seed hardness, and cultivation conditions (Lin et al., 2009; Zhang et al., 2009). A complete understanding of dormancy, germination, and seedling formation in grape seeds is lacking, and more comprehensive studies are needed for perennial species (Leida et al., 2010; Graeber et al., 2012). Indeed, large differences were found between crosses, with the progeny of cv Italia showing only minor losses, while the progeny of varieties, such as VICTORIA and RED GLOBE, reached 74.8%. Heavy losses (up to 59%) were also observed in seedlings in the first stages of growth, especially in the progeny of cv. CARDINAL, BOLGAR REZY, MOLDOVA, and PIZZUTELLA, while the progeny of cv. ITALIA, SOURI, and SACRA ROSSA were hardly affected. Here, too, diseases, unsuitable growing conditions, and transplanting methods can have a major influence on the establishment rate of the seedlings, but the variety also seems to play a decisive role (Butiuc and Coste, 2023). Overall, the Italia variety provided very encouraging results. Italia is a late-maturing table grape bred in 1911 from a cross of BICANE × MUSCAT AMBURG. It is the most important Apulian variety and is known for its large bunches and tasty, crunchy berries, as well as its good transportability and storability (Torres et al., 2017). The good performance observed makes this variety a good candidate for table grape breeding programs.

The early application of MAS led to the identification of 7,696 seedless putative plants (49% of the total progeny) of which 79% were confirmed in the field trials indicating a good efficiency of the marker *p3_VvAGL11* and its usefulness in long-term, labor-intensive, and expensive grapevine selection programs (Bergamini et al., 2013). However, the lower predictive power of the marker observed in the CORNIOLA and AP25 crosses suggests that alternative/additional modifications at the associated *196-bp* locus may be involved in the determinism of seedlessness. A single nucleotide modification in the *VvAGL11* gene has been shown to be responsible for seedlessness in *V. vinifera* (Royo et al., 2018; Ocarez et al., 2020). However, it is very likely that other recombination events and SNPs in the coding region, with small but stable effects, are involved in the complex genetic architecture of apyrenia leading to the failure of the marker (Ocarez et al., 2020; Li et al., 2015). Further characterization of the seedlessness locus and further genetic analyses will help to clarify the reasons for the failure of the marker’s predictive power in crosses with these varieties.

Regarding the berry colors of the progeny, recombination was observed in most crosses. Such variability is not surprising in table grape, a crop in which crosses between different varieties are widespread resulting in extensive gene recombination. In grapevine, differences in berry color are generally due to somatic mutations associated with the VvMybA gene family, which is highly polymorphic and determines the variation in anthocyanin content in berries (Lijavetzky et al., 2006; Fournier-Level et al., 2009; Carbonell-Bejerano et al., 2017; Ferreira et al., 2020; Röckel et al., 2022). Here, too, it is likely that minor loci also play a role, which would explain the controversial results especially for the varieties BLACK MAGIC, MOSCATO, and RED GLOBE. On the other hand, these varieties seem to be a very good resource for increasing color variability in table grape breeding programs. As far as the berry flavor trait is concerned, half of the crosses between neutral female and muscadine male resulted in 49% of neutral offspring, 18% in aromatic offspring, and 34% in partly aromatic and partly neutral progeny. In grapevine, monoterpenes are the key compounds responsible for the Muscat flavor, and a major QTL was co-localized with the 1-deoxy-D-xylulose 5-phosphate synthase (*VvDXS*) gene, encoding for the 1-deoxy-D-xylulose 5-phosphate synthase enzyme, which is involved in the plastidial pathway of terpene biosynthesis (Battilana et al., 2009; Emanuelli et al., 2010; Li et al., 2023). Our results are difficult to interpret, and further genetic studies of this trait and its heritability will be carried out in the future.

The crosses carried out to obtain the pyramiding of seedlessness and resistance to DM and PM resulted in 230 putative resistant seedless genotypes, 20 of which were resistant to both diseases. The preliminary field observations yielded results that were generally consistent with molecular selection with offspring showing medium-to-high scores of resistance. However, some inconsistencies were also recorded, which makes further evaluation crucial in the coming years. It will also be necessary to extend the analysis to other resistance alleles involved in PM such as *Ren3* and *Ren9* (Zini et al., 2019). The pedigree of parental varieties, such as PÖLÖSKEI MUSKOTÁLY, TERÉZ, and PALATINA, created in Hungary in 1957 from back-crosses of Seyve-Villard and other French–American hybrids, lack information on the origin of their resistance and should be integrated. This would help to establish an efficient protocol for the early identification of resistance genes in *V. vinifera*, and would facilitate the lengthy and costly process of grapevine breeding (Hoffmann et al., 2007; Tóth-Lencsés et al., 2015).

## 5 Conclusions

In recent years, a more sustainable and environmentally friendly management of table grape production has also become possible through the use of resistant varieties. The use of multiple sources of resistance and MAS facilitates the pyramiding of key resistance genes. The breeding program described here has enabled the selection of 10 new selections (**Supplementary Figure 1**) that combine seedlessness with phenotypically important commercial traits such as color and aroma. These selections are in the process of patent registration in the National Register of Vine Varieties. Others will be the subject of an evaluation plan in the coming years, which will lead to the selection of varieties that meet the ever-changing requests of producers and consumers, also with regard to the new diseases that continue to appear in the Mediterranean area, such as *Xylella fastidiosa fastidiosa*, the causal agent of Pierce’s disease, which has recently also appeared in Puglia (https://www.osservatoriofitosanitario.regione.puglia.it/). The availability of new tools, such as genomic selection approaches using genome-wide molecular markers, will certainly be better suited to capture the complex genetic architecture of resistance and other quantitative traits. The implementation of these approaches in grape breeding will provide new opportunities to promote more sustainable and environmentally friendly viticulture.

## Data availability statement

The original contributions presented in the study are included in the article/**Supplementary Material**. Further inquiries can be directed to the corresponding authors.

## Author contributions

MMM: Investigation, Methodology, Validation, Writing – original draft, Writing – review & editing. LP: Data curation, Investigation, Writing – original draft. CP: Data curation, Investigation, Writing – review & editing. VR: Data curation, Writing – review & editing. DB: Investigation, Validation, Writing – review & editing. IM: Visualization, Writing – review & editing. PLN: Validation, Visualization, Writing – review & editing. CM: Funding acquisition, Methodology, Validation, Writing – review & editing, Resources.
