# Supplementary material for: Breeding new seedless table grapevines for a more sustainable viticulture in Mediterranean climate
Source: Front Plant Sci. 2024 Apr 5;15:1379642. doi: 10.3389/fpls.2024.1379642 (PMC11027070; doi:10.3389/fpls.2024.1379642)
Supplement: Supplementary file 1 [file Presentation_1.pptx]

## Slide 1
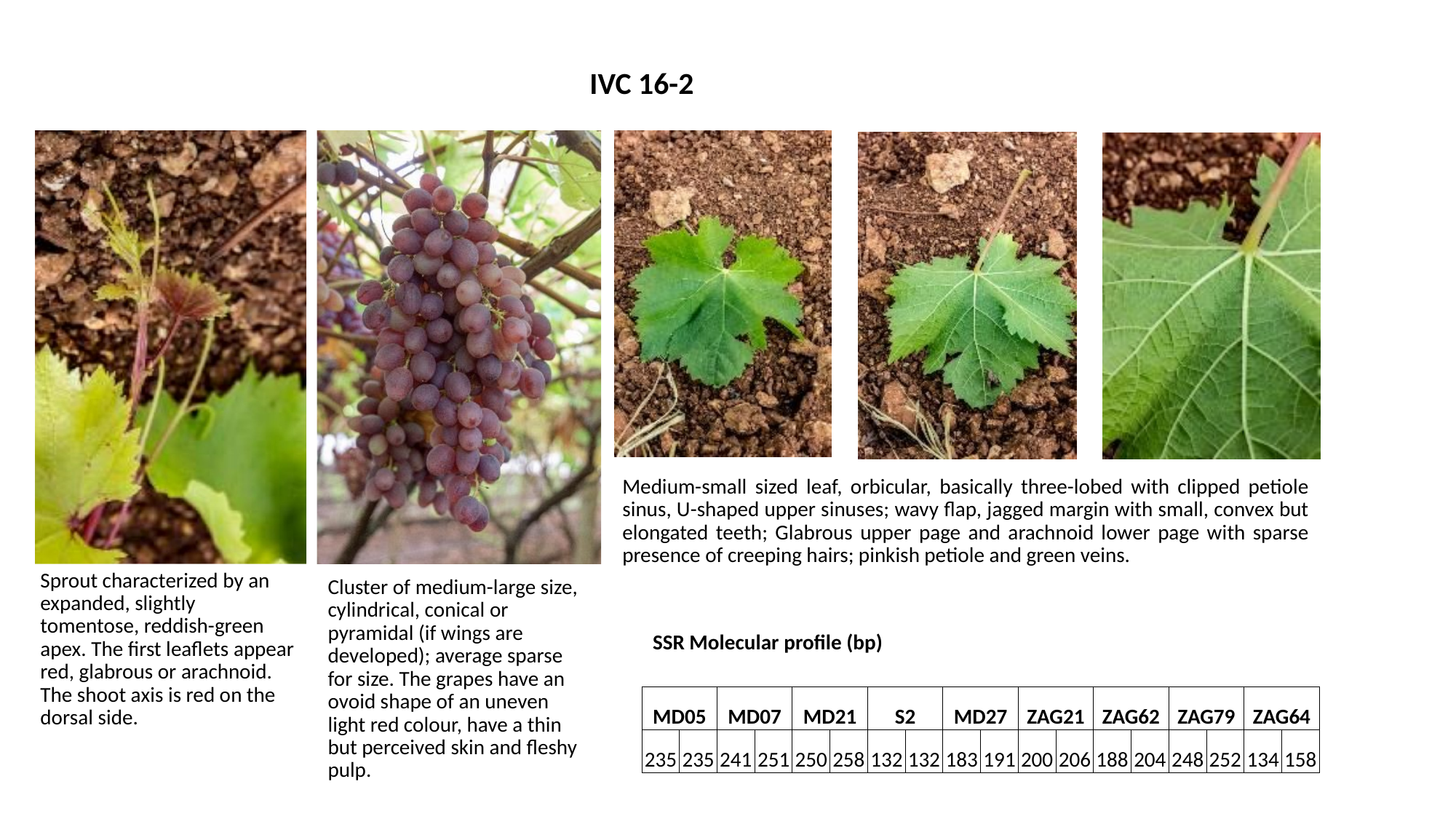

# IVC 16-2
Sprout characterized by an expanded, slightly tomentose, reddish-green apex. The first leaflets appear red, glabrous or arachnoid. The shoot axis is red on the dorsal side.
Medium-small sized leaf, orbicular, basically three-lobed with clipped petiole sinus, U-shaped upper sinuses; wavy flap, jagged margin with small, convex but elongated teeth; Glabrous upper page and arachnoid lower page with sparse presence of creeping hairs; pinkish petiole and green veins.
Cluster of medium-large size, cylindrical, conical or pyramidal (if wings are developed); average sparse for size. The grapes have an ovoid shape of an uneven light red colour, have a thin but perceived skin and fleshy pulp.
SSR Molecular profile (bp)
| MD05 | | MD07 | | MD21 | | S2 | | MD27 | | ZAG21 | | ZAG62 | | ZAG79 | | ZAG64 | |
| --- | --- | --- | --- | --- | --- | --- | --- | --- | --- | --- | --- | --- | --- | --- | --- | --- | --- |
| 235 | 235 | 241 | 251 | 250 | 258 | 132 | 132 | 183 | 191 | 200 | 206 | 188 | 204 | 248 | 252 | 134 | 158 |

## Slide 2
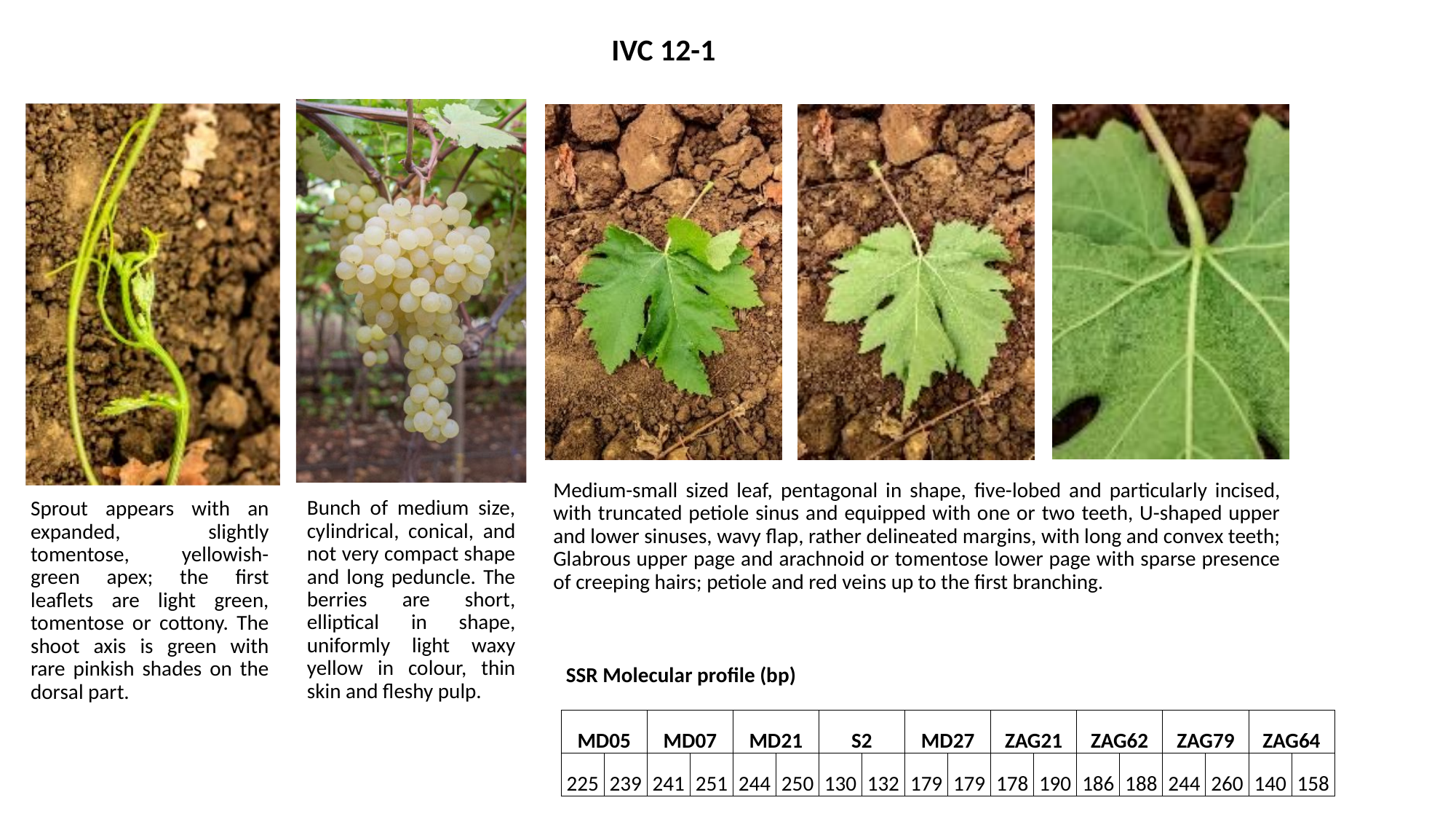

IVC 12-1
Sprout appears with an expanded, slightly tomentose, yellowish-green apex; the first leaflets are light green, tomentose or cottony. The shoot axis is green with rare pinkish shades on the dorsal part.
Medium-small sized leaf, pentagonal in shape, five-lobed and particularly incised, with truncated petiole sinus and equipped with one or two teeth, U-shaped upper and lower sinuses, wavy flap, rather delineated margins, with long and convex teeth; Glabrous upper page and arachnoid or tomentose lower page with sparse presence of creeping hairs; petiole and red veins up to the first branching.
Bunch of medium size, cylindrical, conical, and not very compact shape and long peduncle. The berries are short, elliptical in shape, uniformly light waxy yellow in colour, thin skin and fleshy pulp.
SSR Molecular profile (bp)
| MD05 | | MD07 | | MD21 | | S2 | | MD27 | | ZAG21 | | ZAG62 | | ZAG79 | | ZAG64 | |
| --- | --- | --- | --- | --- | --- | --- | --- | --- | --- | --- | --- | --- | --- | --- | --- | --- | --- |
| 225 | 239 | 241 | 251 | 244 | 250 | 130 | 132 | 179 | 179 | 178 | 190 | 186 | 188 | 244 | 260 | 140 | 158 |

## Slide 3
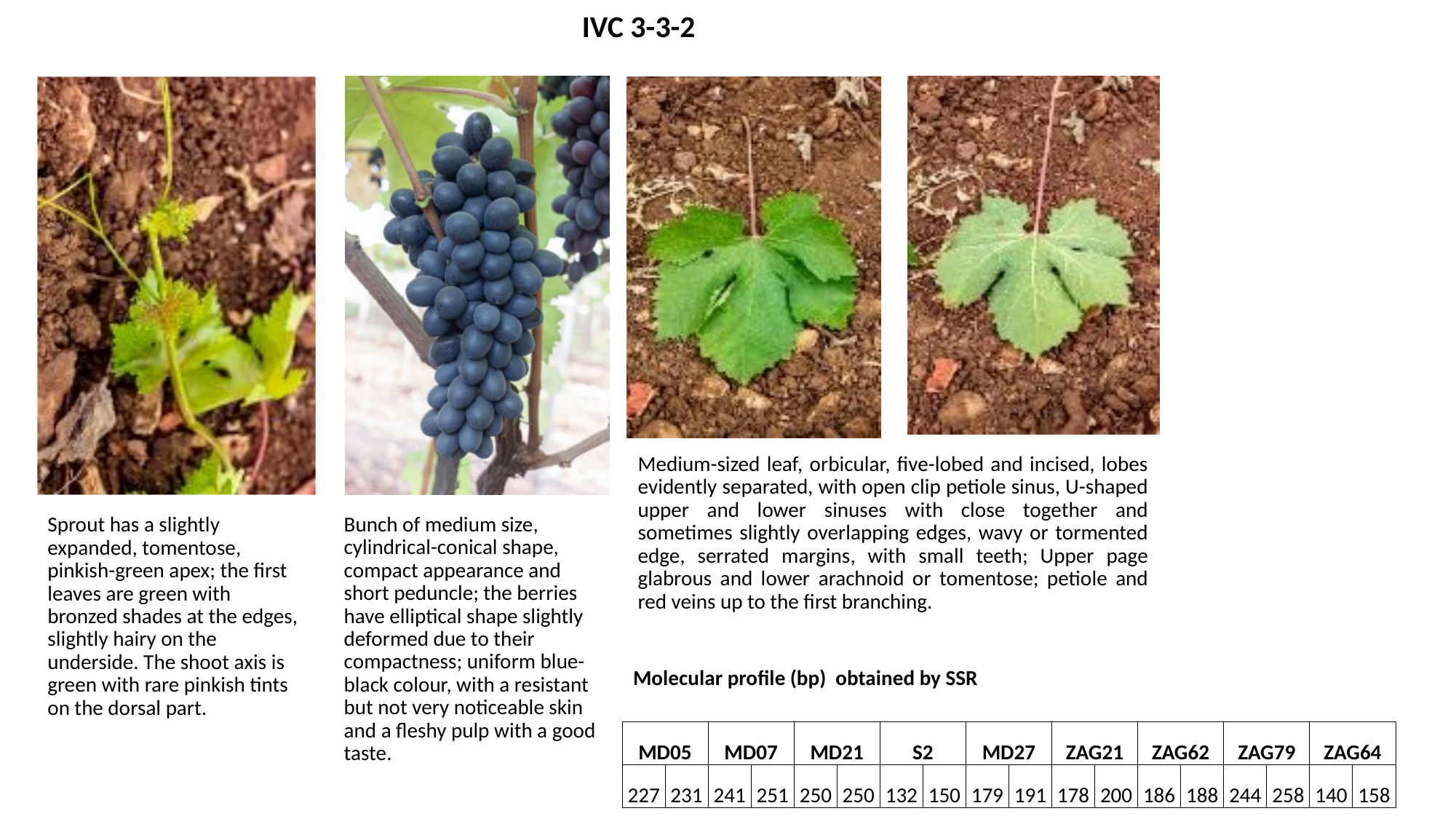

IVC 3-3-2
Medium-sized leaf, orbicular, five-lobed and incised, lobes evidently separated, with open clip petiole sinus, U-shaped upper and lower sinuses with close together and sometimes slightly overlapping edges, wavy or tormented edge, serrated margins, with small teeth; Upper page glabrous and lower arachnoid or tomentose; petiole and red veins up to the first branching.
Sprout has a slightly expanded, tomentose, pinkish-green apex; the first leaves are green with bronzed shades at the edges, slightly hairy on the underside. The shoot axis is green with rare pinkish tints on the dorsal part.
Bunch of medium size, cylindrical-conical shape, compact appearance and short peduncle; the berries have elliptical shape slightly deformed due to their compactness; uniform blue-black colour, with a resistant but not very noticeable skin and a fleshy pulp with a good taste.
Molecular profile (bp) obtained by SSR
| MD05 | | MD07 | | MD21 | | S2 | | MD27 | | ZAG21 | | ZAG62 | | ZAG79 | | ZAG64 | |
| --- | --- | --- | --- | --- | --- | --- | --- | --- | --- | --- | --- | --- | --- | --- | --- | --- | --- |
| 227 | 231 | 241 | 251 | 250 | 250 | 132 | 150 | 179 | 191 | 178 | 200 | 186 | 188 | 244 | 258 | 140 | 158 |

## Slide 4
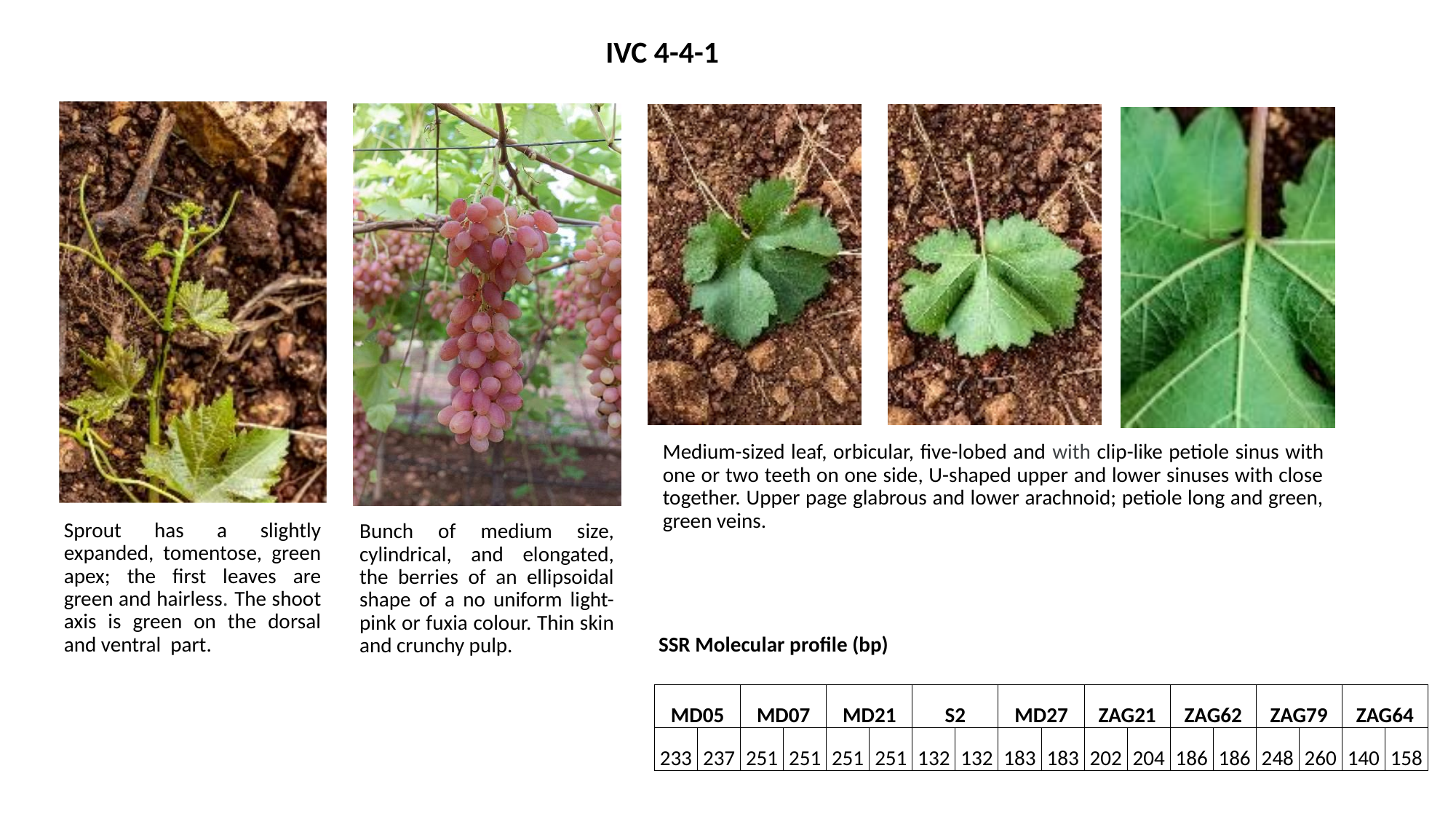

# IVC 4-4-1
Medium-sized leaf, orbicular, five-lobed and with clip-like petiole sinus with one or two teeth on one side, U-shaped upper and lower sinuses with close together. Upper page glabrous and lower arachnoid; petiole long and green, green veins.
Sprout has a slightly expanded, tomentose, green apex; the first leaves are green and hairless. The shoot axis is green on the dorsal and ventral part.
Bunch of medium size, cylindrical, and elongated, the berries of an ellipsoidal shape of a no uniform light-pink or fuxia colour. Thin skin and crunchy pulp.
SSR Molecular profile (bp)
| MD05 | | MD07 | | MD21 | | S2 | | MD27 | | ZAG21 | | ZAG62 | | ZAG79 | | ZAG64 | |
| --- | --- | --- | --- | --- | --- | --- | --- | --- | --- | --- | --- | --- | --- | --- | --- | --- | --- |
| 233 | 237 | 251 | 251 | 251 | 251 | 132 | 132 | 183 | 183 | 202 | 204 | 186 | 186 | 248 | 260 | 140 | 158 |

## Slide 5
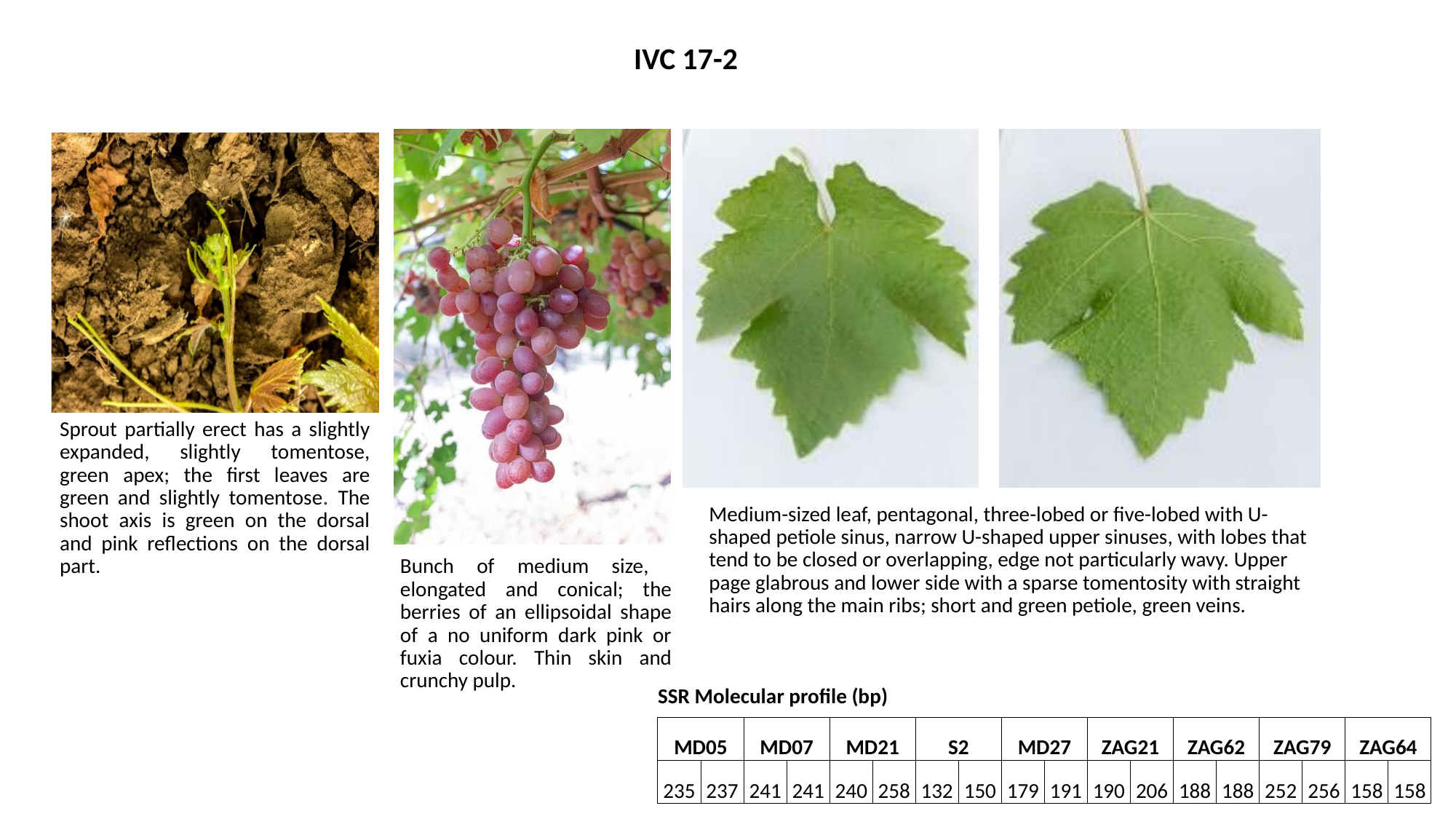

# IVC 17-2
Sprout partially erect has a slightly expanded, slightly tomentose, green apex; the first leaves are green and slightly tomentose. The shoot axis is green on the dorsal and pink reflections on the dorsal part.
Medium-sized leaf, pentagonal, three-lobed or five-lobed with U-shaped petiole sinus, narrow U-shaped upper sinuses, with lobes that tend to be closed or overlapping, edge not particularly wavy. Upper page glabrous and lower side with a sparse tomentosity with straight hairs along the main ribs; short and green petiole, green veins.
Bunch of medium size, elongated and conical; the berries of an ellipsoidal shape of a no uniform dark pink or fuxia colour. Thin skin and crunchy pulp.
SSR Molecular profile (bp)
| MD05 | | MD07 | | MD21 | | S2 | | MD27 | | ZAG21 | | ZAG62 | | ZAG79 | | ZAG64 | |
| --- | --- | --- | --- | --- | --- | --- | --- | --- | --- | --- | --- | --- | --- | --- | --- | --- | --- |
| 235 | 237 | 241 | 241 | 240 | 258 | 132 | 150 | 179 | 191 | 190 | 206 | 188 | 188 | 252 | 256 | 158 | 158 |

## Slide 6
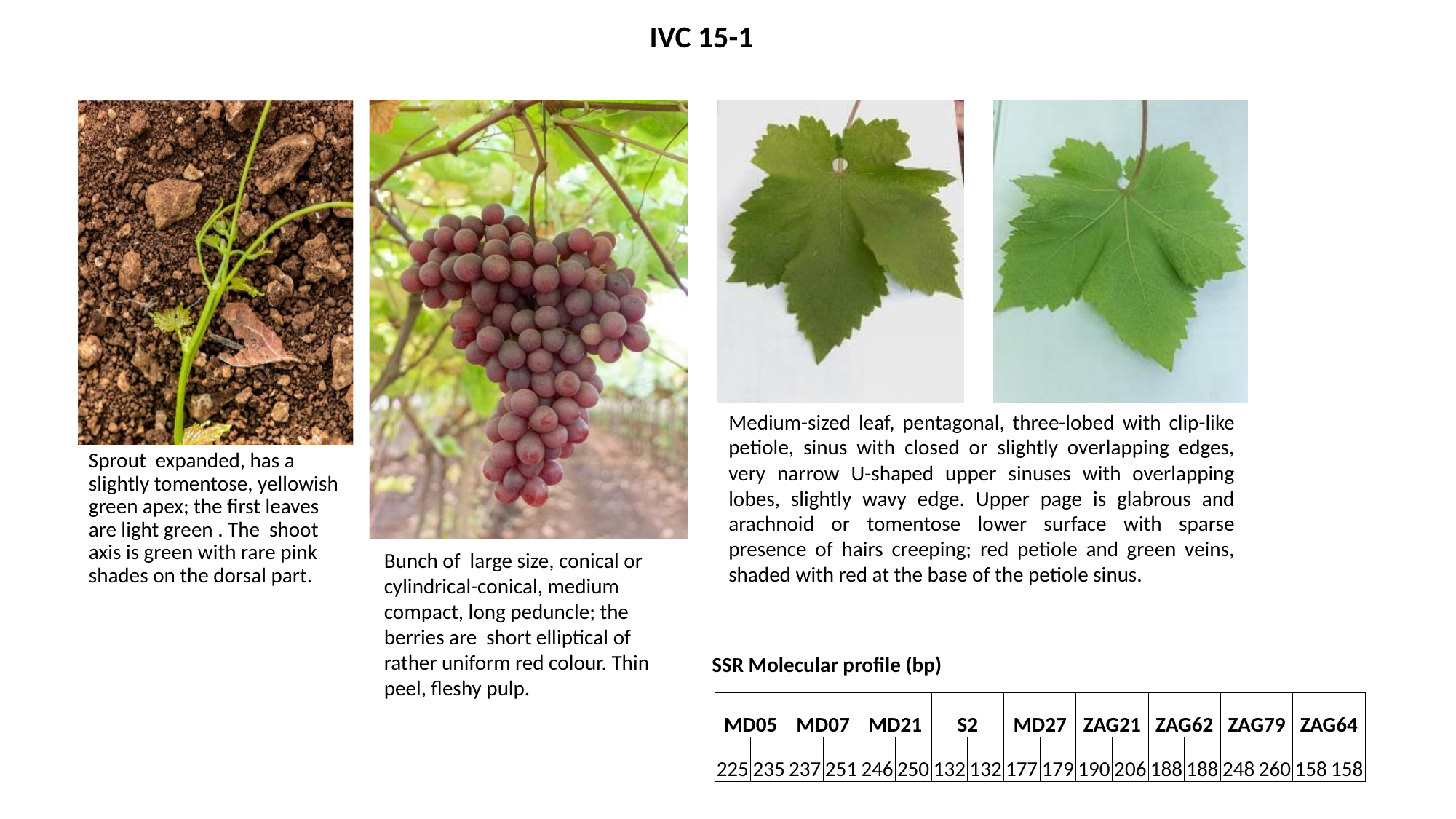

IVC 15-1
Medium-sized leaf, pentagonal, three-lobed with clip-like petiole, sinus with closed or slightly overlapping edges, very narrow U-shaped upper sinuses with overlapping lobes, slightly wavy edge. Upper page is glabrous and arachnoid or tomentose lower surface with sparse presence of hairs creeping; red petiole and green veins, shaded with red at the base of the petiole sinus.
Sprout expanded, has a slightly tomentose, yellowish green apex; the first leaves are light green . The shoot axis is green with rare pink shades on the dorsal part.
Bunch of large size, conical or cylindrical-conical, medium compact, long peduncle; the berries are short elliptical of rather uniform red colour. Thin peel, fleshy pulp.
SSR Molecular profile (bp)
| MD05 | | MD07 | | MD21 | | S2 | | MD27 | | ZAG21 | | ZAG62 | | ZAG79 | | ZAG64 | |
| --- | --- | --- | --- | --- | --- | --- | --- | --- | --- | --- | --- | --- | --- | --- | --- | --- | --- |
| 225 | 235 | 237 | 251 | 246 | 250 | 132 | 132 | 177 | 179 | 190 | 206 | 188 | 188 | 248 | 260 | 158 | 158 |

## Slide 7
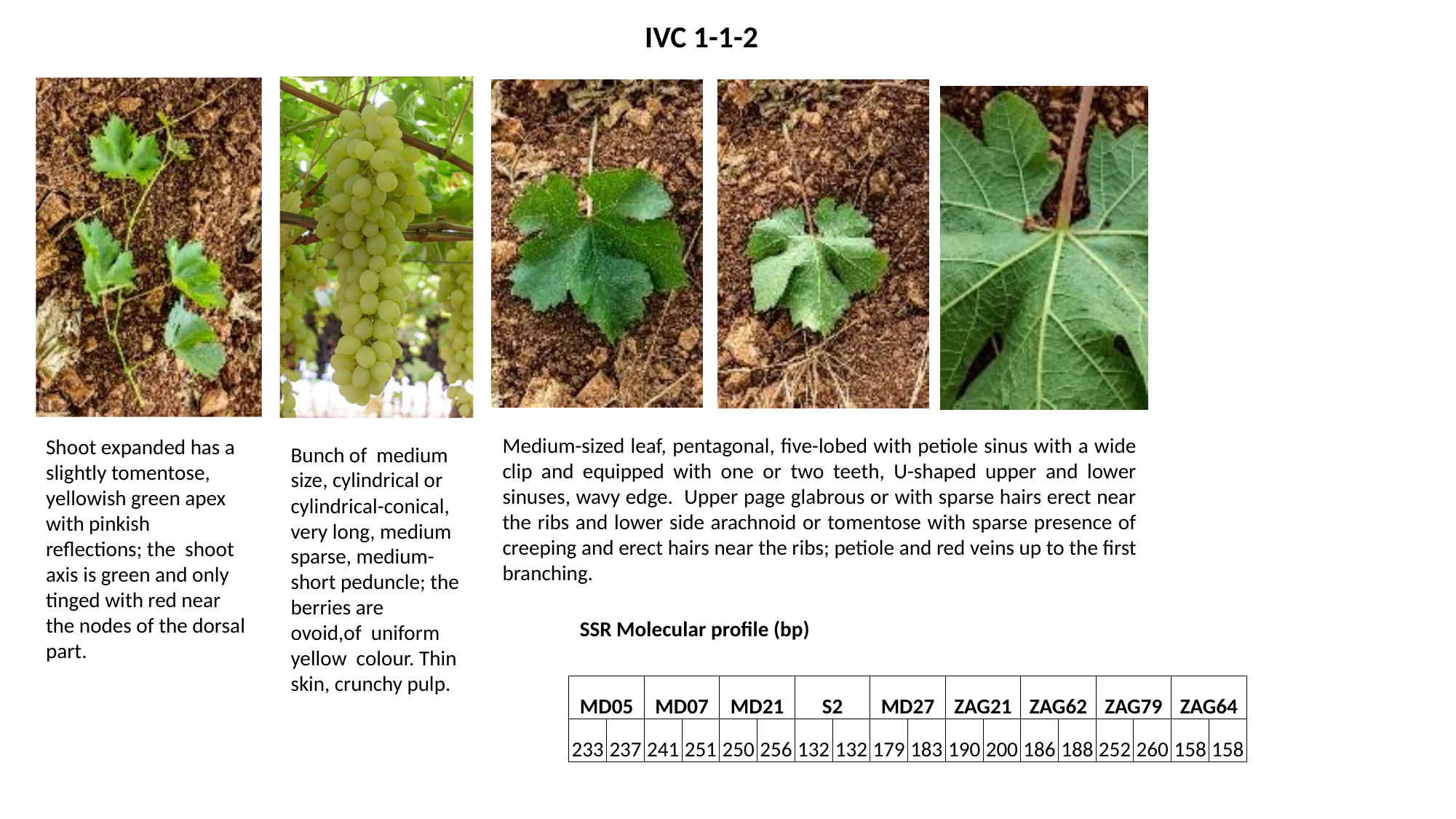

IVC 1-1-2
Medium-sized leaf, pentagonal, five-lobed with petiole sinus with a wide clip and equipped with one or two teeth, U-shaped upper and lower sinuses, wavy edge. Upper page glabrous or with sparse hairs erect near the ribs and lower side arachnoid or tomentose with sparse presence of creeping and erect hairs near the ribs; petiole and red veins up to the first branching.
Shoot expanded has a slightly tomentose, yellowish green apex with pinkish reflections; the shoot axis is green and only tinged with red near the nodes of the dorsal part.
Bunch of medium size, cylindrical or cylindrical-conical, very long, medium sparse, medium-short peduncle; the berries are ovoid,of uniform yellow colour. Thin skin, crunchy pulp.
SSR Molecular profile (bp)
| MD05 | | MD07 | | MD21 | | S2 | | MD27 | | ZAG21 | | ZAG62 | | ZAG79 | | ZAG64 | |
| --- | --- | --- | --- | --- | --- | --- | --- | --- | --- | --- | --- | --- | --- | --- | --- | --- | --- |
| 233 | 237 | 241 | 251 | 250 | 256 | 132 | 132 | 179 | 183 | 190 | 200 | 186 | 188 | 252 | 260 | 158 | 158 |

## Slide 8
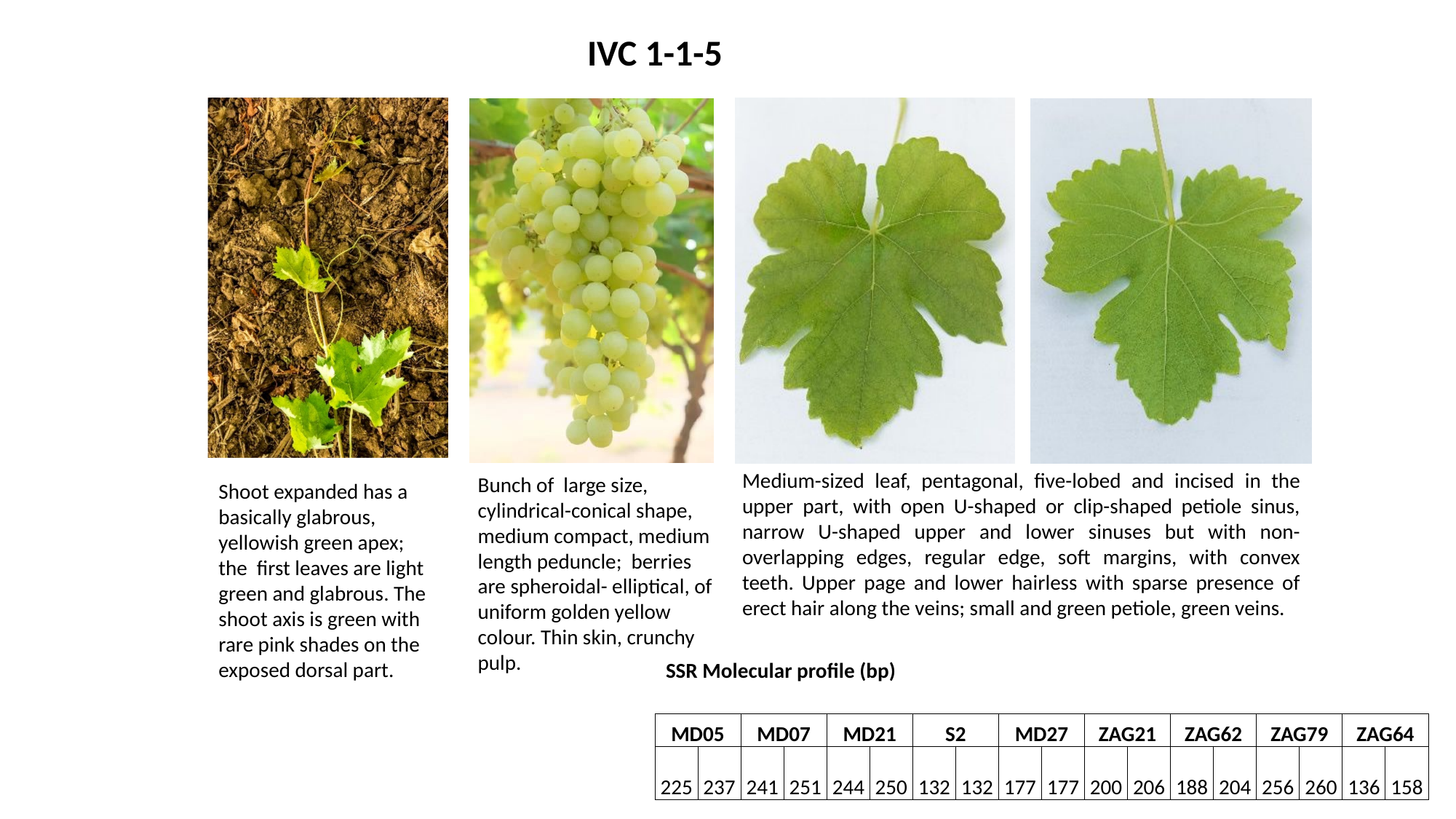

# IVC 1-1-5
Medium-sized leaf, pentagonal, five-lobed and incised in the upper part, with open U-shaped or clip-shaped petiole sinus, narrow U-shaped upper and lower sinuses but with non-overlapping edges, regular edge, soft margins, with convex teeth. Upper page and lower hairless with sparse presence of erect hair along the veins; small and green petiole, green veins.
Bunch of large size, cylindrical-conical shape, medium compact, medium length peduncle; berries are spheroidal- elliptical, of uniform golden yellow colour. Thin skin, crunchy pulp.
Shoot expanded has a basically glabrous, yellowish green apex; the first leaves are light green and glabrous. The shoot axis is green with rare pink shades on the exposed dorsal part.
SSR Molecular profile (bp)
| MD05 | | MD07 | | MD21 | | S2 | | MD27 | | ZAG21 | | ZAG62 | | ZAG79 | | ZAG64 | |
| --- | --- | --- | --- | --- | --- | --- | --- | --- | --- | --- | --- | --- | --- | --- | --- | --- | --- |
| 225 | 237 | 241 | 251 | 244 | 250 | 132 | 132 | 177 | 177 | 200 | 206 | 188 | 204 | 256 | 260 | 136 | 158 |

## Slide 9
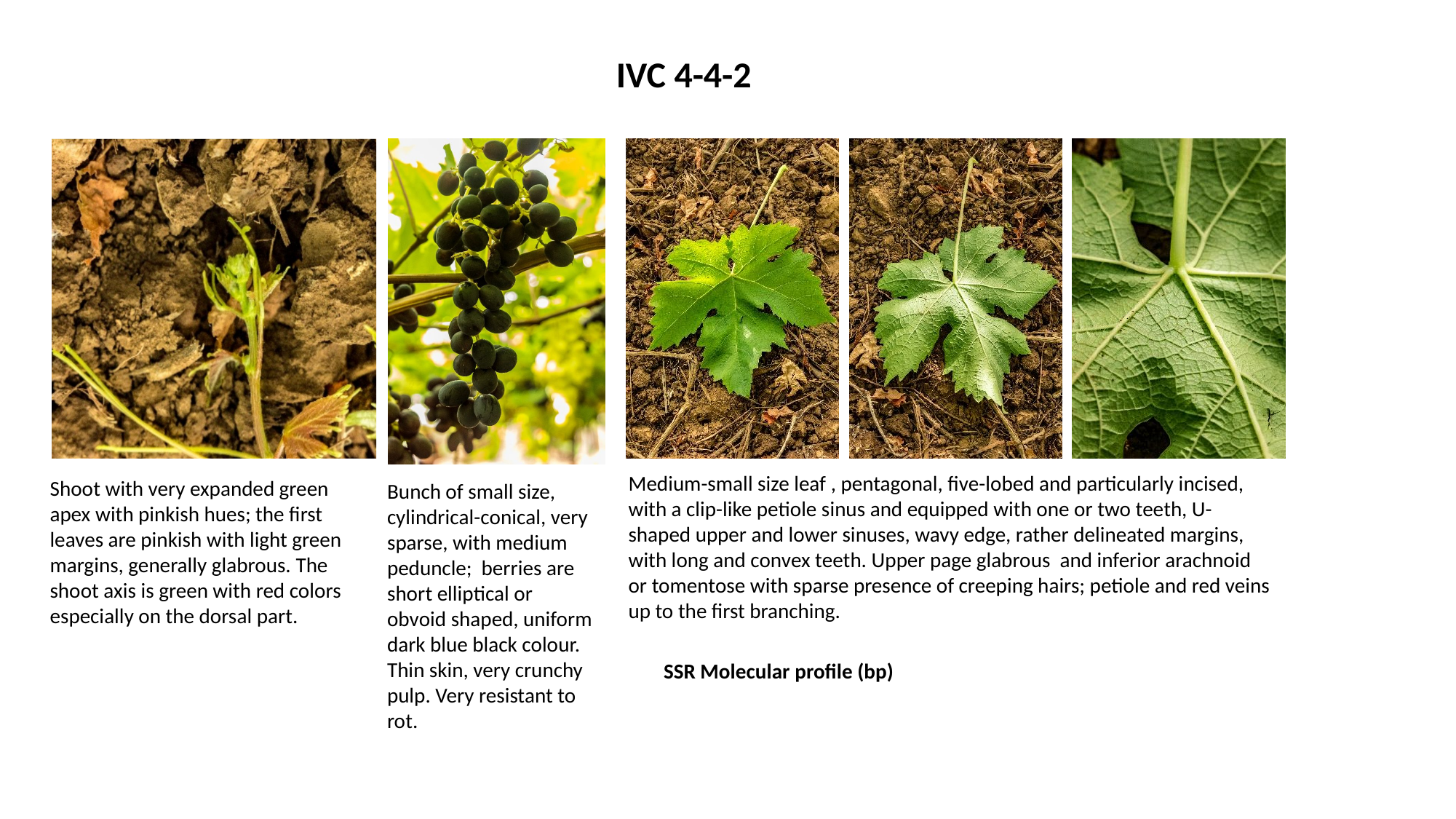

# IVC 4-4-2
Medium-small size leaf , pentagonal, five-lobed and particularly incised, with a clip-like petiole sinus and equipped with one or two teeth, U-shaped upper and lower sinuses, wavy edge, rather delineated margins, with long and convex teeth. Upper page glabrous and inferior arachnoid or tomentose with sparse presence of creeping hairs; petiole and red veins up to the first branching.
Shoot with very expanded green apex with pinkish hues; the first leaves are pinkish with light green margins, generally glabrous. The shoot axis is green with red colors especially on the dorsal part.
Bunch of small size, cylindrical-conical, very sparse, with medium peduncle; berries are short elliptical or obvoid shaped, uniform dark blue black colour. Thin skin, very crunchy pulp. Very resistant to rot.
SSR Molecular profile (bp)

## Slide 10
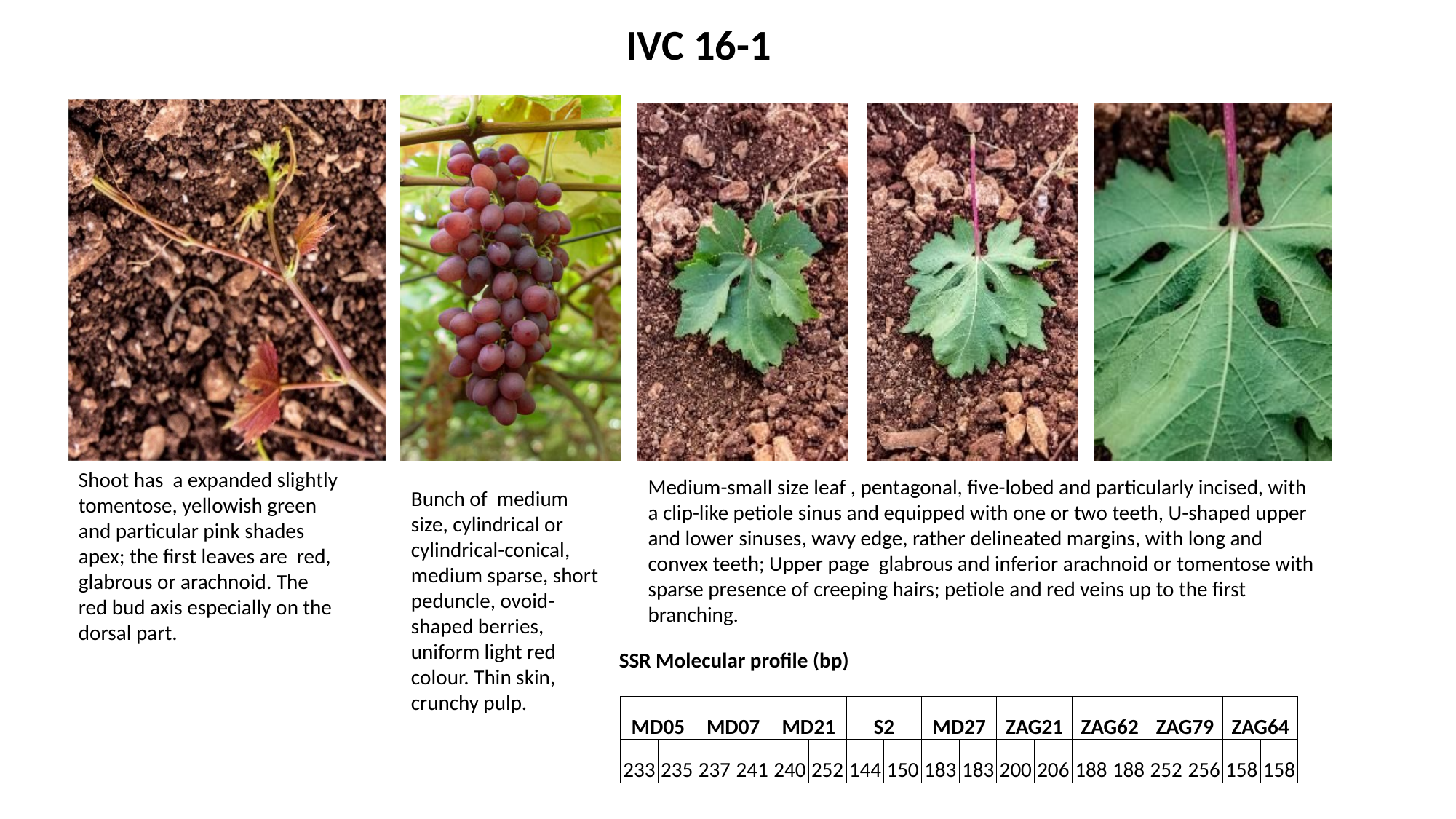

IVC 16-1
Shoot has a expanded slightly tomentose, yellowish green and particular pink shades apex; the first leaves are red, glabrous or arachnoid. The red bud axis especially on the dorsal part.
Medium-small size leaf , pentagonal, five-lobed and particularly incised, with a clip-like petiole sinus and equipped with one or two teeth, U-shaped upper and lower sinuses, wavy edge, rather delineated margins, with long and convex teeth; Upper page glabrous and inferior arachnoid or tomentose with sparse presence of creeping hairs; petiole and red veins up to the first branching.
Bunch of medium size, cylindrical or cylindrical-conical, medium sparse, short peduncle, ovoid-shaped berries, uniform light red colour. Thin skin, crunchy pulp.
SSR Molecular profile (bp)
| MD05 | | MD07 | | MD21 | | S2 | | MD27 | | ZAG21 | | ZAG62 | | ZAG79 | | ZAG64 | |
| --- | --- | --- | --- | --- | --- | --- | --- | --- | --- | --- | --- | --- | --- | --- | --- | --- | --- |
| 233 | 235 | 237 | 241 | 240 | 252 | 144 | 150 | 183 | 183 | 200 | 206 | 188 | 188 | 252 | 256 | 158 | 158 |
